# The Prevalence of Antibiotic-Resistant *Staphylococcus aureus* Nasal Carriage among Industrial Hog Operation Workers, Community Residents, and Children Living in Their Households: North Carolina, USA

**DOI:** 10.1289/EHP35

**Published:** 2016-10-18

**Authors:** Sarah M. Hatcher, Sarah M. Rhodes, Jill R. Stewart, Ellen Silbergeld, Nora Pisanic, Jesper Larsen, Sharon Jiang, Amanda Krosche, Devon Hall, Karen C. Carroll, Christopher D. Heaney

**Affiliations:** 1 Department of Environmental Sciences and Engineering, UNC Gillings School of Global Public Health, Chapel Hill, North Carolina, USA; 2 Department of Environmental Health and Engineering, Johns Hopkins Bloomberg School of Public Health, Baltimore, Maryland, USA; 3 Microbiology and Infection Control, Statens Serum Institute, Copenhagen, Denmark; 4 Rural Empowerment Association for Community Help, Warsaw, North Carolina, USA; 5 Johns Hopkins University School of Medicine, Baltimore, Maryland, USA; 6 Department of Epidemiology, Johns Hopkins Bloomberg School of Public Health, Baltimore, Maryland, USA

## Abstract

**Background:**

Antibiotic use in industrial hog operations (IHOs) can support the emergence of antibiotic-resistant (ABR) *Staphylococcus aureus*. The extent of ABR *S. aureus* exposure in IHO workers and children living in their households remains unclear.

**Objective:**

We investigated ABR *S. aureus* nasal carriage prevalence among adults with versus without occupational exposure to IHOs and among children living in their households.

**Methods:**

In total, 198 IHO worker–child household pairs and 202 community referent (CR) adult–child household pairs completed a questionnaire and provided a nasal swab which was analyzed for *S. aureus,* methicillin-resistant *S. aureus* (MRSA), multidrug-resistant *S. aureus* (MDRSA), absence of *scn* (putative marker of livestock association), and *spa* type.

**Results:**

*S. aureus* nasal carriage prevalence was higher among IHO (53%) compared with CR (31%) adults [adjusted prevalence ratio (aPR): 1.40; 95% confidence interval (CI): 1.07, 1.83], but MRSA nasal carriage prevalence was uncommon (2–3%) in IHO and CR adults. MDRSA nasal carriage prevalence was similar among IHO workers and CR adults (12% vs. 8%; aPR: 1.14; 95% CI: 0.56, 2.29). Nasal carriage prevalence was higher among IHO compared with CR children for *S. aureus* (49% vs. 31%; aPR: 1.50; 95% CI: 1.13, 1.99), MRSA (14% vs. 6%; aPR: 2.37; 95% CI: 1.14, 4.92), and MDRSA (23% vs. 8%; aPR: 2.64; 95% CI: 1.47, 4.75). We also found suggestive evidence of a higher prevalence of *S. aureus*, MRSA, and MDRSA among children living with an IHO worker who did versus did not report taking personal protective equipment (PPE) home from the IHO. Livestock-associated *S. aureus* nasal carriage predominated among IHO workers.

**Conclusion:**

Our findings support the importance of further research on the prevalence and potential sources of exposure to ABR *S. aureus* among children living with IHO workers.

**Citation:**

Hatcher SM, Rhodes SM, Stewart JR, Silbergeld E, Pisanic N, Larsen J, Jiang S, Krosche A, Hall D, Carroll KC, Heaney CD. 2017. The prevalence of antibiotic-resistant *Staphylococcus aureus* nasal carriage among industrial hog operation workers, community residents, and children living in their households: North Carolina, USA. Environ Health Perspect 125:560–569; http://dx.doi.org/10.1289/EHP35

## Introduction

In industrial hog operations (IHOs), low doses of antibiotics are commonly administered in hog feed and water for the purposes of growth promotion and disease prevention (Love et al. 2011; Silbergeld et al. 2008). This practice can promote antibiotic-resistant (ABR) *Staphylococcus aureus*, including methicillin-resistant (MRSA) (de Neeling et al. 2007; Lewis et al. 2008; Smith et al. 2009; Voss et al. 2005) and multidrug-resistant (MDRSA) resistance to ≥ three antibiotic classes (Nadimpalli et al. 2015; Rinsky et al. 2013) strains. ABR *S. aureus* can be transmitted between pigs and persons who have occupational exposure to pigs (e.g., IHO workers, swine veterinarians) (Khanna et al. 2008; Price et al. 2012; Smith et al. 2009; van den Broek et al. 2009). Evidence of human exposure to livestock-associated (LA-) ABR *S. aureus* emerged in the European Union in the early 2000s when a novel clonal complex (CC) of MRSA (CC398) was documented in swine and swine-worker households and subsequently in communities with a high density of swine production (Smith and Pearson 2011). Evidence of human infection due to LA-MRSA CC398 emerged in the European Union via case reports of infections among swine workers and their children, including infants (Hartmeyer et al. 2010; Huijsdens et al. 2006; Voss et al. 2005).

In the United States, knowledge of LA-*S. aureus* exposure dynamics has progressed more slowly than in the European Union. MRSA CC398 has been detected in swine and swine workers in the Midwestern United States (Smith et al. 2009) and North Carolina (Nadimpalli et al. 2015; Rinsky et al. 2013), but little is known about *S. aureus* exposure dynamics between swine workers and their children. Filling this knowledge gap is critical because children’s (particularly young children’s and infants’) frequent mouthing and fomite touching behaviors (Ko et al. 2007; Mattioli et al. 2015) and developing immune systems (Goenka and Kollmann 2015) may enhance their susceptibility to *S. aureus* exposure and infection (Chen et al. 2009; Fridkin et al. 2005; Graber et al. 2011). Moreover, the proportion of children colonized with community-associated (CA-) MRSA strains in pediatric intensive care units (PICUs) appears to be increasing over time, increasing from 6.7% in 2001 to 36.1% in 2009 in one PICU (Hermos et al. 2013). Known risk factors for child exposure to and infection with CA-*S. aureus* strains include child care attendance, household crowding, pet ownership, sports participation, and parental or caregiver occupation in a health care or child care setting (Fritz et al. 2008; Miller et al. 2011; Nakamura et al. 2002; Shapiro et al. 2009). However, to our knowledge, parental or caregiver occupation in IHOs has not been investigated as a risk factor of child *S. aureus* nasal carriage and infection in the United States.

Here, we investigated nasal carriage of ABR *S. aureus* among children living with IHO workers in the United States. We examined prevalence of *S. aureus*, MRSA, and MDRSA nasal carriage outcomes, including several putative markers of LA-*S. aureus*—*scn*-negative *S. aureus*, *S. aureus* CC398, and *S. aureus* CC9 (Nadimpalli et al. 2015; Rinsky et al. 2013; Ye et al. 2016a, 2016b)—among adults with occupational exposure to IHOs, adult community resident without occupational exposure to IHOs, and one child < 7 years of age living in the same household as each adult in rural southeastern North Carolina.

## Methods

### Ethics Statement

This study was a collaboration among the Johns Hopkins Bloomberg School of Public Health (JHSPH), the Rural Empowerment Association for Community Help (REACH) and the University of North Carolina at Chapel Hill (UNC). The JHSPH Institutional Review Board (IRB) approved this study (IRB00004608). The UNC Non-Biomedical IRB approved reliance on the JHSPH IRB. Before participation, adult participants provided written informed consent. Parents or legal guardians provided written informed assent for their participating child < 7 years of age and provided questionnaire responses for their participating child.

### Study Population

Data were collected between March and October 2014 in North Carolina by community organizers from REACH and researchers from JHU and UNC. Using a snowball sampling approach, community organizers recruited both IHO and community referent (CR) participants residing anywhere in the top 10 hog-producing counties in North Carolina, the second-largest hog-producing state in the United States (USDA and NCDA&CS 2010). In order of highest to lowest density of hogs, these counties were Duplin, Sampson, Bladen, Wayne, Robeson, Greene, Pender, Lenoir, Jones, and Columbus (USDA and NCDA&CS 2010). One adult (≥ 18 years old) worker and one child (< 7 years of age) were recruited from each household. If there was more than one child (< 7 years of age) living in the household, the children’s parent or legal guardian was asked to choose which child would participate and provide permission and assent for the child to participate. IHO households were those with at least one adult who was employed full time at an IHO (IHO group) at the time of the study visit or within the prior 3 months and did not have contact with other livestock at work (e.g., poultry). CR households were those with adult residents who were not employed in any livestock-related industry within the prior 12 months. Study participants were ineligible if *a*) they or their household members worked in a health care or child care setting (defined as being cared for at an establishment or home along with five or more unrelated children); *b*) no child < 7 years of age lived in the household; or *c*) they could not respond to a questionnaire in English or Spanish. Eligibility criteria were assessed during recruitment and before beginning questionnaire data and sample collection. Data analysis included eligibility criteria assessment; ineligible participants were not included in the analysis.

### Questionnaire Data and Nasal Swab Collection

Questionnaire responses and nasal swabs were collected during the same study visit. Adult participants and parents or legal guardians of child participants reported occupational activities and workplace exposures, personal and household member contact with livestock and pets, environmental exposures (e.g., known nearby land application of livestock or human waste), health care exposures (defined as having been to a hospital, doctor’s office, or other medical facility for any reason), child care attendance (defined as being cared for at an establishment or home along with five or more unrelated children), medical history, and demographic information. The study questionnaires were developed in collaboration with REACH organizers who obtained feedback from current and former IHO workers and community residents living in the top 10 hog-producing counties of North Carolina about the appropriate phrasing and formatting of questions. The IHO group questionnaire included more detailed questions about hog and other livestock production activities than the CR group questionnaire. During training sessions, REACH community organizers were trained to consistently apply study eligibility criteria and were trained as interviewers to administer the questionnaires to participants to account for varying literacy levels. REACH interviewers included those who were fluent in English and Spanish. Participants had to have the ability to respond to study questions in English or Spanish; adult participants responded for any child participants who were too young to respond. Study personnel obtained a nasal swab from adult participants by rotating a sterile, double-tipped BD BBL™ CultureSwab™ five times clockwise and five times counter-clockwise in both nares. To minimize discomfort of child participants, two single, mini-tipped BD BBL™ CultureSwab™ nasal swabs were collected in the same manner from child participants. A set of trip blanks were collected before transport from REACH to UNC and stored with samples during transport. Swabs were stored in Stuart’s medium at 4°C and transported to UNC within 5 days of sample collection for processing.

### Detection of *S. aureus* and MRSA

One tip of the double-tipped adult swab and one of the mini-tipped children’s swabs were aseptically clipped into 1 mL sterile 0.01 M phosphate buffered saline (PBS). After vortexing for 1 min, 100 μL were pipetted directly onto CHROMagar™ Staph aureus (CA) media (BD, Franklin Lakes, NJ) and distributed using a sterilized spreader and an inoculating turntable. Plates were then incubated at 37°C for 24 hr, and up to two colonies with morphological characteristics of *S. aureus* were picked and streaked to isolation on CA media. If fewer than two colonies grew on CA after 24 hr, the entire PBS swab suspension including swab was inoculated into 10 mL Mueller–Hinton broth supplemented with 6.5% NaCl and incubated overnight at 37°C. This enrichment was then streaked on CA and Baird–Parker plates and incubated at 37°C for 24 hr and 48 hr, respectively (Nadimpalli et al. 2013). Up to two presumptive *S. aureus* colonies were then archived at –80°C in brain–heart infusion broth with 15% glycerol until further characterization. Catalase and tube coagulase testing with rabbit plasma (BD BBL™, Franklin Lakes, NJ) were used to confirm *S. aureus* biochemical characteristics before molecular testing.

### *S. aureus* and MRSA Molecular Confirmation

Following crude DNA extraction (Reischl et al. 2000), a multiplex polymerase chain reaction (PCR) assay was employed to detect the *S. aureus*-specific gene *spa*, as well as *mecA*, *mecC* (*mecA*LGA251), *scn*, and *pvl* [39] as described by Stegger et al. (2012). A sequenced *S. aureus* isolate obtained from a previous study (Rinsky et al. 2013) was used as the positive control for *spa*, *mec*A, *scn*, and *pvl*, and LGA251 (García-Álvarez et al. 2011) was used for *mec*C. Each PCR product was confirmed by gel electrophoresis on a 3% agarose gel. Among morphologically and biochemically characteristic *S. aureus* isolates that lacked *spa* by multiplex PCR, we attempted to amplify alternate *S. aureus*-specific genes by PCR including an alternate *spa* target (DTU Food 2009), *nuc* (Poulsen et al. 2003) and *fem*A (Paule et al. 2004). Finally, matrix-assisted laser desorption ionization time-of-flight mass spectrometry (MALDI-TOF MS) was performed on 12 isolates that were negative for both *spa* primers but positive for *nuc* or *fem*A to confirm *S. aureus* identity. From archived cultures, isolates were streaked on Tryptic Soy Agar with 5% Sheep Blood and incubated overnight at 37°C. MALDI-TOF MS was performed using the FDA-cleared VITEK MS per manufacturer’s recommendations for direct colony spotting (bioMerieux, Durham, NC) (Rychert et al. 2013).

Isolates that met the following criteria were classified as *S. aureus*: *a*) *spa*-positive; *b*) positive for *nuc* and *fem*A; or *c*) identified as *S. aureus* by MALDI-TOF-MS. Confirmed *S. aureus* isolates were classified as MRSA if they were positive for *mec*A or *mec*C by PCR (“MRSA genotype”).

### Antibiotic Susceptibility of *S. aureus*

Confirmed *S. aureus* isolate(s) from each positive nasal swab were tested for susceptibility to the following 12 classes of antibiotics: aminoglycosides, beta-lactams, cephalosporins, fluoroquinolones, lincosamides, macrolides, oxazolidinones, rifamycin, streptogramins, sulfonamide, nitrofuran, and tetracyclines (see Table S1). The Kirby–Bauer disk diffusion method was used to test each isolate’s susceptibility to all antibiotic classes. Interpretation of zones of inhibition was reported as susceptible, resistant, or intermediately resistant (where applicable) according to the Clinical and Laboratory Standards Institute guidelines (CLSI 2015). In erythromycin-resistant isolates, inducible clindamycin resistance was assessed using the D-zone test (Steward et al. 2005). *S. aureus* isolates that exhibited complete phenotypic resistance to at least three antibiotic classes were classified as MDRSA (Magiorakos et al. 2012). *S. aureus* isolates that exhibited complete resistance to oxacillin were classified as phenotypic MRSA (“MRSA phenotype”). MRSA isolates meeting the definition of MDRSA were classified as multidrug-resistant MRSA.

### S. aureus spa *Typing*

Staphylococcal protein A (*spa*) typing was performed by amplifying the *spa* gene as described above. PCR products were sequenced by Eton Biosciences, Inc. (Research Triangle Park, NC). *spa* types were assigned using the Ridom Staph Type standard protocol (http://www.ridom.com) and the Ridom SpaServer (http://spa.ridom.de/index.shtml). To assess nasal carriage prevalence of CC398 and CC9, *spa* types were assigned to these clonal complexes based on the scientific literature and analyses of *S. aureus* isolates collected from our prior livestock worker studies in North Carolina (Nadimpalli et al. 2015; Rinsky et al. 2013). The *spa* types classified as CC398 and CC9 are listed in the Supplemental Material in “Assignment of *spa* types to clonal complexes (CCs).” For within household concordance, *spa* types were assigned to multi-locus sequence types (MLSTs) based on the scientific literature, analyses of *S. aureus* isolates collected from our prior livestock worker studies in North Carolina (Nadimpalli et al. 2015; Rinsky et al. 2013), and queries of the Ridom SpaServer.

### *S. aureus* Nasal Carriage Outcomes

We examined the following nasal carriage outcomes: *S. aureus*, MRSA, MDRSA, *S. aureus* CC398, *S. aureus* CC9, and *scn*-negative *S. aureus*, MRSA, and MDRSA. An individual was considered positive for a given *S. aureus* nasal carriage outcome if either of their two *S. aureus* isolates met the criteria for that outcome. We report MRSA prevalence by two criteria: *a*) *S. aureus* that was positive for *mecA* or *mecC* by PCR (hereafter, MRSA genotype) and *b*) *S. aureus* that exhibited complete resistance to oxacillin by Kirby–Bauer disk diffusion (hereafter, MRSA phenotype).

### Statistical Analysis

We first compared the distribution of demographic characteristics and potential risk factors for *S. aureus* nasal carriage among the IHO and CR groups for adult and child participants, respectively. Next, we calculated the crude prevalence of *S. aureus* nasal carriage outcomes in each exposure group (IHO and CR) for adults and children, respectively. For *S. aureus* outcomes for which there was sufficient sample size, we used log-binomial regression models to calculate crude prevalence ratios (PRs) and 95% confidence intervals (CIs) comparing nasal carriage prevalence for each *S. aureus* outcome in the IHO versus the CR groups among adults and children, respectively. We examined potential confounding covariates for which there was sufficient sample size (≥ 5) within strata of the exposure and *S. aureus* nasal carriage outcome and *a priori* evidence that a covariate could be acting as a confounder. The following covariates were considered as potential confounders in adjusted log-binomial regression models: adult household member’s highest level of education [< high school or ≥ high school, used as a proxy for household socioeconomic status (SES)], total number of individuals living in the household (tertiles categorized as 2–3, 4, or ≥ 5 people, used as a proxy for household crowding), and household pet ownership (defined as owning a pet of any kind kept in- or outside the household). Race/ethnicity and sex were imbalanced between the IHO and CR groups, leading to small sample sizes within strata of the exposure, which precluded inclusion of these covariates in adjusted regression models. Other potential confounding covariates (recent antibiotic use, gym attendance, and contact sports within the previous 3 months, smoker living in the household, and child care attendance) were uncommon (sample size of < 5 within strata of exposure and *S. aureus* nasal carriage outcome) in our study population, and thus were not included as covariates in log-binomial regression models. Based on IHO workers’ responses to the study questionnaire, we summarized the distribution of characteristics of the IHOs where they worked and their IHO work activities, including size of the IHO, number of pigs each IHO worker typically contacted per day, pig life stage, hours worked per week, length of employment at current IHO, and whether the IHO worker took personal protective equipment (PPE) home from the IHO, performed cleaning activities at work using disinfectants (ammonia or Clorox bleach), and handled dead pigs. These factors are categorized as shown in Table S2. We estimated PR (95% CI) to examine whether any IHO characteristics and IHO work activities were associated with *S. aureus* nasal carriage outcomes of IHO children. All statistical analyses were completed in SAS version 9.4 (SAS Institute Inc., Cary, NC).

## Results

### Participant Characteristics

A total of 400 households participated in this study, comprised of 198 IHO and 202 CR households (Table 1). The majority of IHO participants resided in Duplin (54%) and Sampson (37%) counties, and the majority of CR participants resided in Duplin (41%) and Wayne (40%) counties (Table 1). Overall, IHO adults and children were older than CR adults and children (Table 1). In the IHO group, roughly half of adult IHO participants were female (46%), whereas 80% of adult CR participants were female. The majority of adults in the IHO group self-identified as Hispanic (94%). The majority of adults in the CR group self-identified as non-Hispanic (67%)—a majority of non-Hispanic CR participants self-identified as black alone (125/202; 62%). Few other racial/ethnic groups were represented in the CR group (8/202 white alone, not Hispanic; 3/202 multi-race/ethnicity). The majority of adult participants in both groups reported that their household’s primary health care provider was a private doctor or clinic. However, more IHO (34%) than CR (21%) adults reported not having any health insurance. In general, fewer IHO adult participants reported antibiotic use, healthcare contact, and gym attendance in the 3 months before sample collection compared with CR adult participants. Specifically, 45% of CR adults reported visiting a health care facility in the 3 months before sample collection, compared with only 5% of adults in the IHO group. CR adult participants reported less participation in contact sports within 3 months of sample collection (Table 1).

**Table 1:** Description of study population characteristics among industrial hog operation (IHO) and community referent (CR) participants, North Carolina, 2014 [*n* (%)].

Characteristic	Adults		Children	
	IHO	CR	IHO	CR
Age (years)	198	202	198	202
0–2	—	—	32 (16)	75 (37)
3–5	—	—	116 (59)	100 (50)
6–< 7	—	—	50 (25)	27(13)
18–27	47 (24)	108 (54)	—	—
28–37	88 (44)	62 (31)	—	—
38–47	42 (21)	24 (12)	—	—
≥ 47	21 (11)	8 (4)	—	—
Sex	198	202	198	202
Male	107 (54)	41 (20)	123 (62)	90 (45)
Female	91 (46)	161 (80)	75 (38)	112 (54)
Race/ethnicity^*a*^	198	202	198	202
Any Hispanic	186 (94)	66 (33)	187 (94)	68 (34)
Non-Hispanic	12 (6)	136 (67)	11 (6)	134 (66)
Hispanic alone	185 (93)	66 (33)	186 (94)	65 (32)
Black alone, not Hispanic	12 (6)	125 (62)	11 (6)	122 (60)
White alone, not Hispanic	0 (0)	8 (4)	0 (0)	3 (1)
Multi-race/ethnicity	1 (< 1)	3 (2)	1 (< 1)	12 (6)
Black/Hispanic	0 (0)	0 (0)	0 (0)	2 (1)
White/Hispanic	1 (< 1)	0 (0)	1 (< 1)	1 (< 1)
Black/white	0 (0)	1 (< 1)	0 (0)	6 (3)
American Indian/white	0 (0)	1 (< 1)	0 (0)	0 (0)
Black/American Indian/white	0 (0)	1 (< 1)	0 (0)	3 (1)
Education	198	201	—	—
< High school	139 (70)	44 (22)	—	—
≥ High school	59 (30)	157 (78)	—	—
County of residence	198	202	198	202
Duplin	106 (54)	83 (41)	106 (54)	83 (41)
Sampson	73 (37)	24 (12)	73 (37)	24 (12)
Wayne	9 (5)	81 (40)	9 (5)	81 (40)
Bladen	5 (3)	11 (5)	5 (3)	11 (5)
Pender	4 (2)	1 (< 1)	4 (2)	1 (< 1)
Lenoir	0 (0)	2 (1)	0 (0)	2 (1)
Greene	1 (< 1)	0 (0)	1 (< 1)	0 (0)
Primary healthcare provider	198	202	197	202
Private doctor or clinic	147 (74)	150 (74)	165 (84)	177 (88)
Emergency room	13 (7)	15 (7)	14 (7)	6 (3)
Hospital	9 (5)	13 (6)	1 (< 1)	5 (3)
Free clinic	21 (11)	14 (7)	7 (4)	6 (3)
Urgent care	1 (< 1)	4 (2)	3 (2)	1 (< 1)
Company clinic or doctor	6 (3)	3 (1)	3 (2)	3 (1)
Other	0 (0)	1 (< 1)	0 (0)	4 (2)
Do not use healthcare	1 (< 1)	2 (1)	4 (2)	0 (0)
Health insurance	198	202	198	202
None	67 (34)	42 (21)	11 (6)	7 (3)
Company/employer	79 (40)	19 (9)	39 (20)	12 (6)
Private	2 (1)	8 (4)	0 (0)	5 (2)
Public	49 (25)	133 (66)	147 (74)	178 (88)
Other	1 (< 1)	0 (0)	1 (< 1)	0 (0)
Total number of household members (tertiles)	198	196	198	196
2–3	69 (35)	106 (54)	69 (35)	106 (54)
4	65 (33)	39 (20)	65 (33)	39 (20)
≥ 5	64 (32)	51 (26)	64 (32)	51 (26)
Household pet^*b*^	198	201	198	201
No	133 (67)	162 (81)	133 (67)	162 (81)
Yes	65 (33)	39 (19)	65 (33)	39 (19)
Pet location	64	39	64	39
Indoor only	4 (6)	10 (26)	4 (6)	10 (26)
Outdoor only	49 (76)	21 (54)	49 (76)	21 (54)
Indoor and outdoor	11 (17)	8 (21)	11 (17)	8 (21)
Smoker^*c*^	198	202	—	—
No	167 (84)	130 (64)	—	—
Yes	31 (16)	72 (36)	—	—
Recent antibiotic use^*d*^	198	202	198	202
No	196 (99)	185 (92)	196 (99)	192 (95)
Yes	2 (1)	17 (8)	2 (1)	10 (5)
Recent healthcare contact^*d*^^,^^*e*^	198	202	198	202
No	188 (95)	112 (55)	173 (87)	115 (57)
Yes	10 (5)	90 (45)	25 (13)	87 (42)
Contact sports^*d*^^,^^*f*^	198	202	198	202
No	186 (94)	200 (99)	196 (99)	201 (> 99)
Yes	12 (6)	2 (1)	2 (1)	1 (< 1)
Gym attendance^*d*^^,^^*g*^	198	202	—	—
No	193 (97)	176 (87)	—	—
Yes	5 (3)	26 (13)	—	—
Child care attendance^*d*^^,^^*h*^	—	—	198	202
No	—	—	193 (97)	179 (89)
Yes	—	—	5 (3)	23 (11)
^***a***^Any Hispanic includes participants whose self-reported race/ethnicity was Hispanic alone or Hispanic and one or more of the following: black, white, Asian, Pacific Islander, American Indian, or other. Non-Hispanic includes individuals whose self-reported race/ethnicity was one or more of the following: black, white, Asian, Pacific Islander, American Indian, or other. ^***b***^Defined as owning a pet of any kind kept in- or outside the household. ^***c***^Defined as ever smoked cigarettes or having regularly used cigarillos, cigars, or pipes for ≥ 6 months. ^***d***^Within 3 months of sample collection. ^***e***^Defined as having been to a hospital, doctor’s office, or other medical facility for any reason. ^***f***^Defined as playing any recreational sport during which human contact is common (e.g., soccer). In questionnaire, phrasing was “contact sport.” ^***g***^Defined as attending a workout/sports gym or facility. ^***h***^Defined as being cared for at an establishment or home along with five or more unrelated children.				

Roughly half of participating children in the CR group were female, whereas only 38% of children in the IHO group were female. The majority of children in the IHO group were identified as Hispanic by their assenting caregiver (94%), whereas in the CR group the majority of children were identified as non-Hispanic (66%). The vast majority of children in this study had public health insurance (e.g., Medicaid). Caregivers reported that few children used antibiotics within 3 months before sample collection; however, more children within the CR group (42%) reported visiting a health care facility in the 3 months before sample collection, compared with children in the IHO group (13%). Reported child care attendance was also higher among CR children (11%) compared with IHO children (3%) (Table 1).

### *S. aureus*, MRSA, and MDRSA Nasal Carriage Prevalence among Adults and Children

The prevalence of *S. aureus* nasal carriage was significantly higher among IHO adults (53%) than among CR adults (31%) (PR = 1.68; 95% CI: 1.32, 2.15), including after adjustment for highest level of education of adult participant, total number of individuals living in the home, and the presence of pets in the home [adjusted PR (aPR) = 1.40; 95% CI: 1.07, 1.83] (Table 2). Prevalence of MRSA genotype nasal carriage was similar between IHO adults (2%) and CR adults (4%) (Table 2). The prevalence of MRSA genotype (28/198) compared with MRSA phenotype (27/198) was in agreement for all participants except for one IHO child participant. The *S. aureus* isolate from this child was genotypically but not phenotypically resistant. Prevalence of MDRSA nasal carriage was higher among IHO adults (12%) compared to CR adults (8%), but the crude and adjusted point estimates had wide confidence intervals (Table 2). A complete description of antibiotic resistance characteristics, *scn* gene status, and *spa* type for individual *S. aureus* isolates recovered from adult and child *S. aureus* carriers is provided in Excel File Table S1.

**Table 2:** Prevalence of *S. aureus*, MRSA, and MDRSA in the industrial hog operation (IHO) compared to the community referent (CR) group among adult and child study participants, North Carolina, 2014.

Nasal carriage outcome	Adults			Children		
	No. pos/total (%)	Crude PR (95% CI)	Adjusted aPR (95% CI)^*a*^	No. pos/total (%)	Crude PR (95% CI)	Adjusted aPR (95% CI)^*a*^
*S. aureus*
IHO	104/198 (53)	1.68 (1.32, 2.15)	1.40 (1.07, 1.83)	97/198 (49)	1.60 (1.24, 2.05)	1.50 (1.13, 1.99)
CR	63/202 (31)	Reference	Reference	62/202 (31)	Reference	Reference
MRSA genotype^*b*^
IHO	4/198 (2)	—	—	28/198 (14)	2.38 (1.25, 4.55)	2.37 (1.14, 4.92)
CR	7/202 (3)	Reference	Reference	12/202 (6)	Reference	Reference
MRSA phenotype^*c*^
IHO	4/198 (2)	—	—	27/198 (14)	2.30 (1.20, 4.40)	2.07 (1.02, 4.23)
CR	7/202 (3)	Reference	Reference	12/202 (6)	Reference	Reference
MDRSA
IHO	24/198 (12)	1.53 (0.84, 2.79)	1.14 (0.56, 2.29)	45/198 (23)	2.87 (1.68, 4.90)	2.64 (1.47, 4.75)
CR	16/202 (8)	Reference	Reference	16/202 (8)	Reference	Reference
Note*:* CI, confidence interval; MRSA, methicillin-resistant *S. aureus*; MDRSA, multidrug-resistant *S. aureus* defined as complete resistance to three or more antibiotic drug classes; pos, positive; PR, prevalence ratio. Dash (—) indicates prevalence ratio not estimated due to low prevalence (< 5%). Complete antibiotic resistance profiles for *S. aureus* isolates from participant carriers are available in Excel File Table S1. ^***a***^Adjusted PR (95% CI) estimated from log-binomial regression model including covariates for level of education of adult participant (< high school, ≥ high school), total number of individuals living in the home (2–3, 4, ≥ 5 people) and the presence of any pets inside or outside of the home. ^***b***^Defined as *mec*A or *mec*C positivity by PCR. ^***c***^Defined as complete resistance to oxacillin.						

*S. aureus* nasal carriage prevalence was 49% among children in the IHO group and 31% among children in the CR group (Table 2). Among children living with IHO workers, 14% and 23% carried MRSA and MDRSA intranasally, respectively, compared with 6% and 8% of CR children (Table 2). Adjusting for covariates, nasal carriage prevalence of *S. aureus*, MRSA, and MDRSA among IHO children was 1.50 (95% CI: 1.13, 1.99), 2.37 (95% CI: 1.14, 4.92), and 2.64 (95% CI: 1.47, 4.75) times that of CR children, respectively (Table 2).

### Prevalence of Livestock-Associated *S. aureus* Nasal Carriage Outcomes among Adults and Children

Among IHO adults, 13% carried *scn*-negative *S. aureus*, 1% carried *scn*-negative MRSA, and 10% carried *scn*-negative MDRSA intranasally, compared with 2%, 0%, and 1% of CR adults, respectively (Table 3). Nasal carriage prevalence of *S. aureus* CC398 was 4% among IHO adults compared with 1% among CR adults, and 6% of IHO adults carried *S. aureus* CC9 intranasally compared with 1% of CR adults. All *S. aureus* CC398 and CC9 carried intranasally by IHO and CR adult participants were *scn*-negative (Table 3).

**Table 3:** Prevalence of *S. aureus* with putative markers of livestock association in the industrial hog operation (IHO) compared to the community referent (CR) group among adult and child participants, North Carolina, 2014.

Nasal carriage outcome	Adults		Children	
	No. pos/total	Prevalence (%)	No. pos/total	Prevalence (%)
*scn*-negative
*S. aureus*
IHO	25/198	13	7/198	4
CR	5/202	2	4/202	2
MRSA^*a*^
IHO	2/198	1	1/198	1
CR	0/202	0	0/202	0
MDRSA^*b*^
IHO	19/198	10	5/198	3
CR	3/202	1	1/202	1
CC398^*c*^
IHO	7/198	4	2/198	1
CR	1/202	1	2/202	1
CC9^*d*^
IHO	12/198	6	1/198	0
CR	2/202	1	1/202	0
Note*:* MDRSA, multidrug-resistant *S. aureus* defined as complete resistance to three or more antibiotic drug classes; MRSA, methicillin-resistant *S. aureus*; pos, positive. Prevalence ratios not estimated due to low prevalences (< 5%). ^***a***^Defined as *mec*A or *mec*C positivity by PCR. ^***b***^Defined as complete resistance to three or more antibiotic drug classes by disk diffusion. ^***c***^*S. aureus* CC398 defined according to the *spa* types listed in the Supplemental Material in “Assignment of *spa* types to clonal complexes (CCs).” All *S. aureus* CC398 carried intranasally by IHO and CR adult participants were *scn*-negative, MDRSA. One of two *S. aureus* CC398 carried intranasally by IHO child participants was *scn*-negative. All *S. aureus* CC398 carried intranasally by CR child participants was *scn*-negative. ^***d***^*S. aureus* CC9 defined according to the *spa* types listed in the Supplemental Material in “Assignment of *spa* types to clonal complexes (CCs).” All *S. aureus* CC9 carried by IHO and CR adult participants were *scn*-negative. None of the *S. aureus* CC9 carried intranasally by IHO and CR children were *scn*-negative.				

The prevalence of *scn*-negative *S. aureus, scn-*negative MRSA, and *scn*-negative MDRSA was low among children in the IHO group (4%, 1%, and 3%, respectively) and the CR group (2%, 0%, and 1%, respectively) (Table 3). Nasal carriage prevalence of *S. aureus* CC398 and *S. aureus* CC9 was 1% among both IHO and CR children. Nasal carriage of *S. aureus* CC398 was identified in two IHO children and two CR children (Table 3). All of the *S. aureus* CC398 isolates in children were *scn*-negative except for one that was recovered from an IHO child participant (see Excel File Table S1). One CR child and one IHO child carried *S. aureus* CC9, and none of these isolates were *scn*-negative (Table 3).

### *S. aureus spa* Types among Adults and Children

The most prevalent *spa* types observed are listed in descending frequency in Table S3. Among IHO adults who were *S. aureus* nasal carriers, the two most common *spa* types were t1937 (41/98) followed by t337 (CC9, 10/98) (see Table S3). *spa* types belonging to CC398 were detected infrequently among IHO adults—t034 (4/98) was the most common CC398-associated *spa* type among IHO adults (see Tables S3 and S4). Among CR adults who were *S. aureus* nasal carriers, t1937 (10/62) and t688 (ST8, 7/62) were most prevalent. Among children who were *S. aureus* nasal carriers, the most prevalent *spa* types in both groups were t008 (ST8, 30/94 IHO and 15/59 CR children) followed by t688 (ST5, 10/94 IHO and 11/59 CR children). A small number of *S. aureus* nasal carriers had discordant *spa* types between their two *S. aureus* nasal swab isolates, including 6 of 98 IHO adults, 3 of 94 IHO children, 1 of 62 CR adults, and 3 of 59 CR children with *S. aureus* positive nasal swabs (see Table S4 for the *spa* types identified in each pair of isolates).

### Within-Household *S. aureus spa* Type Concordance between Adults and Children

Among 88 households that had both an adult and a child who carried *S. aureus*, we observed 20 households (7 of 198 IHO households and 13 of 202 households) in which adults and children were carrying concordant *S. aureus* strains (Table 4), defined as at least one of the *S. aureus* isolates collected from an adult’s nasal swab having an identical *spa* type as at least one of the *S. aureus* isolates collected from the nasal swab of a child living in the same household. Nineteen of the 20 pairs of *spa* type–concordant *S. aureus* had identical ABR profiles, (Table 4). The majority of concordant *S. aureus* strain pairs in both the IHO and CR groups were *scn*-positive, non-MRSA, and non-MDRSA. However, one IHO and one CR adult–child household pair carried concordant *scn*-negative *S. aureus* in their nose. In the IHO household this *S. aureus* was MRSA and MDRSA and belonged to *spa* type t002 (ST5) (Table 4). In the CR household this *S. aureus* was MDRSA and belonged to *spa* type t034 (ST398) (Table 4). Another adult–child pair in the IHO group carried tetracycline-resistant *S. aureus*; however, both isolates were not MDRSA (because only two antibiotic drug classes were resistant), were *scn*-positive, and the *spa* type (t1077) is not commonly associated with livestock (Table 4; see also Excel File Table S1).

**Table 4:** *S. aureus* isolate characteristics for households in which there was concordance of *S. aureus spa*-types between adult and child participants by industrial hog operation (IHO) and community referent (CR) group, North Carolina, 2014.

Group/household	Participant type	*spa* type	MLST	MRSA	MDRSA	*scn* (–)	Antibiotic-resistance profile
IHO
1	Adult	t189	188				AMP, PEN
	Child	t189	188				AMP, PEN
2	Adult	t1077	—				TET, AMP, PEN
	Child	t1077	—				TET, AMP, PEN
3	Adult	t1937	—				AMP, PEN
	Child	t1937	—				AMP, PEN
4	Adult	t688	5				AMP, PEN
	Child	t688	5				AMP, PEN
5	Adult	t002	5	X	X	X	CRO, CC, ERY, OX, AMP, PEN
	Child	t002	5	X	X	X	CRO, CC, ERY, OX, AMP, PEN
6	Adult	t688	5				AMP, PEN
	Child	t688	5				AMP, PEN
7	Adult	t008	8				AMP, PEN
	Child	t008	8				AMP, PEN
CR
1	Adult	t034	398		X	X	CC, ERY, TET, AMP, PEN
	Child	t034	398		X	X	CC, ERY, TET, AMP, PEN
2	Adult	t688	5				AMP, PEN
	Child	t688	5				AMP, PEN
3	Adult	t015	45				AMP, PEN
	Child	t015	45				AMP, PEN
4	Adult	t688	5				AMP, PEN
	Child	t688	5				AMP, PEN
5	Adult	t008	8	X	X		CIP, CRO, GAT, LVX, OX, AMP, PEN
	Child	t008	8	X	X		CIP, CRO, GAT, LVX, OX, AMP, PEN
6	Adult	t493	—				AMP, PEN
	Child	t493	—				AMP, PEN
7	Adult	t688	5				AMP, PEN
	Child	t688	5				AMP, PEN
8	Adult	t493	—				AMP, PEN
	Child	t493	—				AMP, PEN
9	Adult	t688	5				AMP, PEN
	Child	t688	5				AMP, PEN
10	Adult	t2949	—				AMP, PEN
	Child	t2949	—				AMP, PEN
11	Adult	t088	8	X	X		CRO, CC, ERY, OX, AMP, PEN
	Child	t088	8	X	X		CRO, CC, ERY, OX, AMP, PEN
12	Adult	t185	50				AMP, PEN
	Child	t185	50				AMP, PEN
13	Adult	t089	30				NONE
	Child	t089	30				AMP, PEN
Note: AMP, ampicillin; CC, clindamycin; CIP, ciprofloxacin; CRO, ceftriaxone; ERY, erythromycin; GAT, gatifloxacin; LVX, levofloxacin; MDRSA, multidrug-resistant *S. aureus* defined as complete resistance to three or more antibiotic drug classes; MLST, multi-locus sequence type; MRSA, methicillin-resistant *S. aureus* defined as positive for *mec*A or *mec*C; OX, oxacillin; PEN, penicillin; TET, tetracycline; X, the characteristic is present. Antibiotics listed represent distinct drug classes except the beta-lactams (ampicillin, oxacillin, and penicillin) and the quinolones (ciprofloxacin, levofloxacin, and gatifloxacin). Dashes (—) indicate that *spa* types could not be assigned to MLST based on the scientific literature, analyses of *S. aureus isolates* collected from our prior livestock worker studies in NC (Nadimpalli et al. 2015; Rinsky et al. 2013), or queries of Ridom SpaServer (http://spa.ridom.de/index.shtml).							

### Association Between IHO Work Activities and Child *S. aureus* Nasal Carriage Outcomes

Several IHO work activities were associated with the *S. aureus* nasal carriage outcome of IHO child household members (Table 5). The strongest association observed was for IHO workers who reported taking personal protective equipment home from the IHO. In households with an IHO worker participant who did versus did not report taking personal protective equipment home we observed higher child participant nasal carriage prevalence of *S. aureus* (PR = 1.35; 95% CI: 1.02, 1.81), MRSA (PR = 12.07; 95% CI: 3.78, 38.60), and MDRSA (PR = 2.09; 95% CI: 1.23, 3.55) (Table 5). Other IHO work activities that were associated with *S. aureus* nasal carriage outcomes of child household members included working with nursery pigs, performing cleaning activities at work using disinfectants, and handling dead pigs (Table 5). The other IHO work activities and factors evaluated (listed in Table S2) were null or noninterpretable due to insufficient numbers (data not shown).

**Table 5:** Crude associations between occupational activities of the industrial hog operation (IHO) worker and the *S. aureus*, MRSA, and MDRSA nasal carriage outcomes of their child household member, North Carolina, 2014.

IHO work activity	*S. aureus*		MRSA		MDRSA	
	No. pos/total (%)	Crude PR (95% CI)	No. pos/total (%)	Crude PR (95% CI)	No. pos/total (%)	Crude PR (95% CI)
Take PPE home
Yes	45/78 (58)	1.35 (1.02, 1.81)	25/78 (32)	12.07 (3.78, 38.60)	26/78 (33)	2.09 (1.23, 3.55)
No	48/113 (43)	Reference	2/113 (3)	Reference	18/113 (16)	Reference
Use disinfectant^*a*^
Yes	60/112 (54)	1.22 (0.90, 1.64)	25/112 (22)	6.25 (1.95, 20.01)	34/112 (30)	2.32 (1.25, 4.30)
No	37/84 (44)	Reference	3/84 (4)	Reference	11/84 (13)	Reference
Work with nursery pigs
Yes	22/39 (56)	1.18 (0.86, 1.63)	10/39 (26)	2.23 (1.12, 4.45)	11/39 (28)	1.30 (0.73, 2.33)
No	75/157 (48)	Reference	18/157 (11)	Reference	34/157 (22)	Reference
Handle dead pigs
Yes	74/143 (52)	1.24 (0.87, 1.76)	25/143 (17)	3.21 (1.01, 10.19)	39/143 (27)	2.50 (1.12, 5.57)
No	23/55 (42)	Reference	3/55 (5)	Reference	6/55 (11)	Reference
Note: CI, confidence interval; MDRSA, multidrug-resistant *S. aureus* defined as resistance to three or more antibiotic drug classes by disk diffusion; MRSA, methicillin-resistant *S. aureus*; pos, positive; PPE, personal protective equipment; PR, prevalence ratio. ^***a***^Defined as performing cleaning activities at work using ammonia or Clorox bleach disinfectant.						

## Discussion

To our knowledge, this study provides the first estimates of ABR *S. aureus* nasal carriage prevalence among children < 7 years of age living with IHO workers in the United States, which we observed to be high for *S. aureus* (49%) as well as for MRSA (14%) and MDRSA (23%) compared to a community referent group (31%, 6%, and 8% prevalence, respectively). Other studies of pediatric MRSA nasal carriage in community settings have shown prevalence estimates ranging between < 1% and 7.6% (Creech et al. 2005; Nakamura et al. 2002; Shopsin et al. 2000). Although MRSA nasal carriage prevalence among children < 7 years living in CR households (6%) fell within this range, that of children living in IHO households was roughly twice as high. Our findings differ from a 1.3% MRSA nasal carriage prevalence observed among preschool-age children at 24 child care centers in rural eastern North Carolina and Virginia between 2007 and 2010 (Miller et al. 2011). Children living in IHO households also had a higher prevalence of MDRSA nasal carriage (23%) than children in CR households (8%). Because few studies have investigated nasal carriage of MDRSA in healthy children < 7 years of age, it is unclear how our observed prevalence compares to population-based estimates in the United States. Because children and infants may have enhanced susceptibility to ABR *S. aureus* infection (Adcock et al. 1998; Fridkin et al. 2005; Milstone et al. 2010; Naimi et al. 2003) the higher prevalence of MRSA and MDRSA nasal carriage we observed, particularly among children living in IHO households, requires confirmation in other populations including prevalence in other IHO populations and in a study that would permit more detailed assessment of important covariates, potential confounding and effect modification.

The higher ABR *S. aureus* nasal carriage prevalence among IHO children (14% MRSA carriage and 23% MDRSA carriage) compared with CR children (6% MRSA carriage and 8% MDRSA carriage) is somewhat surprising for several reasons. First, among IHO compared with CR adults, we did not estimate a positive prevalence ratio for MRSA and MDRSA. IHO workers’ MRSA nasal carriage prevalence (2%) was similar to CR adults (3%) and general U.S. adult population estimates (1.5%) (Gorwitz et al. 2008), but lower than that observed in IHO workers in the Midwestern United States (45%; 9/20) (Smith et al. 2009) and Europe (30%; 15/50) (van den Broek et al. 2009). We observed a similarly low prevalence of MRSA nasal carriage among industrial livestock operation workers (3%; 3/99) (Rinsky et al. 2013) and IHO workers (5%; 1/22) (Nadimpalli et al. 2015) in our previous studies in North Carolina. The prevalence of MDRSA nasal carriage among IHO workers and CR adults observed in this study is consistent with findings from other studies in North Carolina and Iowa (Nadimpalli et al. 2015; Neyra et al. 2014; Rinsky et al. 2013; Wardyn et al. 2015). Second, we rarely observed *S. aureus* strains with putative markers of livestock-association (i.e., *scn*-negative, CC398, CC9) among IHO and CR children. Instead, among IHO and CR children, we frequently observed nasal carriage of *S. aureus* that are considered human-associated and commonly observed in community settings (i.e., *scn*-positive, *spa* types t008 [ST8] and t688 [ST5]). Some IHO and CR adults also carried these strains. Interestingly, 41 of 104 *S. aureus* nasal carriage positive IHO workers were carrying *scn*-positive, *spa* type t1937, but only one IHO child and no CR children carried this strain. Together, these observations suggest that IHO children predominantly acquire *S*. *aureus* from other sources than their adult household member’s nares. Future studies should aim at identifying livestock and community reservoirs and exposure sources related to acquisition of distinct *scn*-positive *S. aureus* strains by IHO children and adults.

Within-household *S. aureus spa* type concordance was observed in 7 IHO households and 13 CR households. Most concordant households involved *S. aureus* strains that are commonly associated with humans. In 1 IHO and 1 CR household, the adult–child pair was carrying concordant strains of *scn*-negative MDRSA that belonged to *spa* types t002 (ST5) and t034 (ST398), respectively. These are *spa* types that have been identified in hogs and in humans in direct contact with hogs (Hau et al. 2015; Smith et al. 2013). In the IHO household both the adult’s and the child’s *scn*-negative *S. aureus* t002 (ST5) isolates were multidrug-resistant MRSA. Because lineages of ST5 and ST398 have demonstrated a successful capacity for human colonization and infection in community and hospital settings (Bosch et al. 2016; Hau et al. 2015; Larsen et al. 2015), these instances of within-household *S. aureus* concordance suggest the need for further research of potential human-to-human transmission in households with and without occupational livestock exposure. The potential for bidirectional *S. aureus* transmission between adults and children is also important to consider because the predominant LA-MRSA CC398 clone in the European Union (Bosch et al. 2016) started in humans as a methicillin-susceptible *S. aureus* strain and subsequently developed methicillin resistance via adaptation and selective pressures in livestock (Price et al. 2012).

The prevalence ratios and household strain concordance we observed could have been related to an environmental or community transmission pathway. Because this study was conducted in an IHO-dense region of the United States, environmental or community exposures to ABR *S. aureus*, including to LA-*S. aureus* strains, may occur. Relatively few CR adults and children carried *scn*-negative *S. aureus* (adults, 5/202; children, 4/202) and *scn*-negative MDRSA (adults, 3/202; children, 1/202). But considering the increasing number of MRSA CC398 infections in general community populations living in pig-dense regions of Denmark (Larsen et al. 2015) and high human MRSA CC398 nasal carriage prevalence in the Netherlands (Feingold et al. 2012; van Cleef et al. 2010), additional studies are needed to determine whether residential proximity to IHOs is related to ABR *scn*-negative *S. aureus* nasal carriage in this region of North Carolina.

A hypothesis to consider in future studies is the possibility that IHO children’s ABR *S. aureus* nasal carriage prevalence is related to IHO work activities and take-home exposures. We observed some evidence of this based on the higher prevalence ratios for *S. aureus*, MRSA, and MDRSA nasal carriage comparing children who lived with an IHO worker who did versus workers who did not bring personal protective equipment home from the IHO. Thus, it is possible that the higher ABR *S. aureus* nasal carriage prevalences observed among children in the IHO group are attributable to take-home introductions of ABR *S. aureus* on fomites from the IHO to the household. However, these associations were not observed for LA-*S. aureus* strains.

In addition to being a reservoir for strains of LA-*S. aureus*, the IHO work environment may harbor non-LA MRSA and non-LA MDRSA strains (those lacking any of the putative markers of livestock association we employed). Because we evaluated *S. aureus* carriage at only one anatomical site (anterior nares), it is possible that IHO workers could have carried non-LA MRSA and/or non-LA MDRSA strains on other anatomical sites or on personal clothing, boots, workplace protective equipment, or other fomites, which could lead to introductions into their household environment. Among 27 IHOs in Germany, 74% of IHO worker boot swabs were MRSA positive (Friese et al. 2012), demonstrating that work clothing or protective gear may be an important fomite for take-home introductions of ABR *S. aureus.*

Several study limitations should be considered. First, we did not have access to sample pigs and the environment inside IHOs, nor did we have information about dose, type, quantity, and frequency of antibiotic use at IHOs in the study area or at facilities where the IHO adults were employed. This limited our ability to determine whether the low MRSA nasal carriage prevalence we observed among IHO workers reflects low MRSA prevalence in pigs at IHOs in North Carolina. Lack of access to this information also limited our ability to determine whether the antibiotic resistance profiles of *S. aureus* carried intranasally by study participants reflect antibiotic use patterns in IHOs. We also did not swab other body sites, personal clothing of the workers, workplace protective equipment, household surfaces, other household members, or the proximal environment, which limited the determination of mechanisms of MRSA and MDRSA exposure and transmission among adults and children in our study. Other *S. aureus* diversity and transmission studies demonstrated that there exists within-host and temporal diversity of *S. aureus* strains (Paterson et al. 2015), suggesting that reliance upon the *S. aureus* geno- and phenotypes from two anterior nares isolates per individual may not represent the full complexity of within household transmission dynamics.

Second, the putative markers of LA-*S. aureus* that we employed may have less than optimal specificity and/or temporal stability for *S. aureus* strains that originate in swine raised in IHOs. For example, the *scn* gene has been shown to be highly mobile, with phage-mediated loss in an animal host and insertion in a human host potentially occurring in as little as 4 hr (McCarthy et al. 2014; Thammavongsa et al. 2015). *S. aureus spa* types that circulate in livestock also can evolve quickly and putative LA-*S. aureus* markers based on *spa* types, CCs (e.g., CC398 or CC9) and/or MLSTs may have uncertain specificity depending on regional and microbial selection factors (Bosch et al. 2016; Hau et al. 2015; Lahuerta-Marin et al. 2016; Ye et al. 2016b).

Third, participants in this study were volunteers, recruited using a snowball sampling approach. It is possible that selection bias may have occurred if individuals who enrolled as IHO and CR participants differed in some way from those who did not enroll in our study. For example, the percent of people who identified as belonging to the following race/ethnicity categories was different in the CR group compared with the general population (GP) living in the same counties as CR participants: Hispanic or Latino (33% CR vs. 13% GP), black or African American (62% CR vs. 30% GP), and white (4% CR vs. 66% GP) (Table 1) (U.S. Census Bureau 2015). Therefore, our results may not be generalizable to the general population living in the same counties as our participants. Access to employee rosters and perhaps voter registries (Wardyn et al. 2015) could facilitate sampling of IHO employees and community residents, respectively, and improve the generalizability of future studies.

Finally, we incorporated some SES-related variables in aPRs, but we did not measure country of origin or immigration status. *S. aureus* CC398 has been observed in some individuals of Caribbean nationality (Mediavilla et al. 2012) and some families who immigrated from the Dominican Republic to New York City (Bhat et al. 2009). We are unaware of a biological basis of race/ethnicity being causally related to *S. aureus* nasal carriage, but there could be social or cultural factors in the IHO and CR populations that might have contributed to the differences in ABR *S. aureus* nasal carriage prevalence. The small sample sizes in strata of the race/ethnicity covariate between the IHO and CR groups precluded its inclusion in adjusted regression models, and race/ethnicity-stratified PRs could not rule out confounding or effect measure modification (data not shown).

## Conclusions

Our findings suggest that children < 7 years of age living in households with IHO workers were more likely to carry ABR *S. aureus* intranasally, particularly MRSA and MDRSA, than children living in CR households. Based on unadjusted prevalence ratios, IHO workers’ taking home personal protective equipment was positively associated with nasal carriage of *S. aureus*, MRSA and MDRSA among IHO child household members. Interestingly, IHO children were infrequent nasal carriers of putative LA-*S. aureus* strains. We also observed that CR participants who had no occupational livestock contact but live in counties with a high density of IHOs sometimes carry putative LA-*S. aureus* in their noses. The higher ABR *S. aureus* nasal carriage prevalence estimated among IHO children needs to be confirmed in other populations, and potential pathways and mechanisms of exposure should be more closely studied. Because the first evidence of human infection burden for the LA-MRSA CC398 clone in the European Union emerged via case reports among swine workers and their children (Hartmeyer et al. 2010; Huijsdens et al. 2006; Voss et al. 2005), further studies should be conducted to determine whether the MRSA and MDRSA nasal carriage prevalence in children in IHO worker households in the United States represents a risk factor for infection.

## Supplemental Material

(168 KB) PDF

(35 KB) ZIP
