# Supplementary material for: The Prevalence of Antibiotic-Resistant Staphylococcus aureus Nasal Carriage among Industrial Hog Operation Workers, Community Residents, and Children Living in Their Households: North Carolina, USA
Source: Environ Health Perspect. 2016 Oct 18;125(4):560–9. doi: 10.1289/EHP35 (PMC5381988; doi:10.1289/EHP35)
Supplement: (168 KB) PDF [file EHP35.s001.acco.pdf]

Environ Health Perspect
DOI: 10.1289/EHP35

Note to readers with disabilities: *EHP* strives to ensure that all journal content is accessible to all readers. However, some figures and Supplemental Material published in *EHP* articles may not conform to 508 standards due to the complexity of the information being presented. If you need assistance accessing journal content, please contact ehponline@niehs.nih.gov. Our staff will work with you to assess and meet your accessibility needs within 3 working days.

Supplemental Material

The Prevalence of Antibiotic-Resistant *Staphylococcus aureus* Nasal Carriage among Industrial Hog Operation Workers, Community Residents, and Children Living in Their Households: North Carolina, USA

Sarah M. Hatcher, Sarah M. Rhodes, Jill R. Stewart, Ellen Silbergeld, Nora Pisanic, Jesper Larsen, Sharon Jiang, Amanda Krosche, Devon Hall, Karen C. Carroll, and Christopher D. Heaney

Table of Contents

Supplemental Methods
Assignment of *spa* types to clonal complexes (CCs)
Supplemental Tables
Table S1. Antibiotics and the concentrations used in antibiotic susceptibility testing
Table S2. Description of industrial hog operation (IHO) characteristics and IHO occupational activities reported by IHO worker participants, North Carolina, 2014
Table S3. *S. aureus spa*-types that occurred at least twice among adults and children (< 7 yr old) in the industrial hog operation (IHO) group and the community referent (CR) group, North Carolina, 2014

Table S4. *S. aureus spa* types for each participant with discordance in *spa* types in their two nasal swab *S. aureus* isolates, North Carolina, 2014

Additional Files

Supplemental Code and Data ZIP File

Excel File Table S1. Heat map of antimicrobial resistance, *scn* gene status, and *spa* type for *S. aureus* isolates for all participants by group.

Supplemental Methods

Assignment of *spa* types to clonal complexes (CCs)

If a confirmed *S. aureus* belonged to one of the following *spa* types, it was classified as CC398: t011, t034, t108, t567, t571, t899, t1184, t1250, t1255, t1446, t1451, t1456, t1457, t1793, t1928, t2011, t2123, t2330, t2346, t2370, t2582, t2876, t2970, t2974, t3075, t3275, t3423, t3625, t3933, t4208, t4652, t4872, t5052, t5095, t5505, t5706, t5838, t5883, t6228, t6575, t6606, t7880, t8588, t8704, t9345, t9418, t9517, t10055, t10150, t10485, t10890, t11241, t11613, t11681, t12116, t12313, t12314, t12841, t13885, t13972, t14075, t14080, t14089, t14151, t14157, t14158, and t14295. The following *spa* types were classified as CC9: t099, t100, t193, t209, t337, t1045, t1236, t1419, t1430, t2112, t2174, t2922, t3270, t3345, t3446, t3474, t3696, t3992, t4780, t6003, t8890, and t14156.

Supplemental Tables

Table S1. Antibiotics and the concentrations used in antibiotic susceptibility testing.

Antibiotic class	Antibiotic	Concentration
Aminoglycosides	Gentamicin	10 µg
β-lactams	Ampicillin	10 µg
	Penicillin	10 units
	Oxacillin	1 µg
Cephalosporins	Ceftriaxone	30 µg
Fluoroquinolones	Ciprofloxacin	5 µg
	Gatifloxacin	5 µg
	Levofloxacin	5 µg
Nitrofuran	Nitrofurantoin	30 µg
Lincosamides	Clindamycin	2 µg
Macrolides	Erythromycin	15 µg
Oxazolidinones	Linezolid	30 µg
Rifamycin	Rifampin	5 µg
Streptogramins	Quinupristin/dalfopristin	15 µg
Sulfonamide/trimethroprim	Sulfamethoxazole/trimethroprim	23.75/1.25 µg
Tetracycline	Tetracycline	30 µg

Table S2. Description of industrial hog operation (IHO) characteristics and IHO occupational activities reported by IHO worker participants, North Carolina, 2014.

IHO work activity	N pos/Total N (%)
**Size of IHO[a] (# pigs)**	
≤1200	67/197 (34)
>1200 to 4050	65/197 (33)
>4050	65/197 (33)
**Total pigs contacted per day[a] (# pigs)**	
≥130	67/198 (34)
>130 to 300	66/198 (33)
>300	65/198 (33)
**Pig life stage at IHO[b]**	
Nursery	39/196 (20)
Finishing	36/196 (18)
Wean or farrow	83/196 (42)
Feeder	11/196 (6)
Breeding pigs	31/196 (16)
**Hours worked per week (hr)**	
>40	181/198 (91)
≤40	17/198 (9)
**Years worked at current IHO (yr, N = 198)**	Median (IQR) 3 (2, 6)
**Take personal protective equipment home**	78/191 (41)
**Perform cleaning activities using disinfectant at IHO[c]**	112/196 (57)
**Handle dead pigs at work**	143/198 (72)

[a] Categorized in tertiles.
[b] Totals do not sum to 100% because some IHOs had multiple pig life stages. Categories were developed based on pig life stage and substantial overlap among individuals who reported working with both sows and boars or both wean and farrow pigs. Additional overlap was observed between the following categories: sow and wean pigs (2 individuals), sow and farrow pigs (1 individual), nursery and finish pigs (1 individual), wean pigs and boars (1 individual), and farrow pigs and boars (1 individual).
[c]Cleaning activities were defined as use of disinfectant (Clorox/Bleach and/or Ammonia), of pressure washing machinery, or of torch/flame.

Table S3. *S. aureus spa*-types that occurred at least twice among adults and children (< 7 yr old) in the industrial hog operation (IHO) group and the community referent (CR) group, North Carolina, 2014.

	***spa* type**[a]	**No. Positive**[b]**/ Total Carriers**	**Percent (%)**
IHO Adults			
	t1937	41/98	42
	t337	10/98	10
	t008	4/98	4
	t688	4/98	4
	t002	3/98	3
	t034	3/98	3
	t233	3/98	3
	t065	2/98	2
	t189	2/98	2
	t1067	2/98	2
	t5595	2/98	2
	NT	3/98	3
IHO Children			
	t008	30/94	32
	t688	10/94	11
	t701	4/94	4
	t002	2/94	3
	t012	3/94	3
	t189	3/94	3
	t018	2/94	2
	t021	2/94	2
	t180	2/94	2
	t346	2/94	2
	t1077	2/94	2
	t3884	2/94	2
	t4710	2/94	2
	t14782	2/94	2
	NT	2/94	2
CR Adults			
	t1937	10/62	16
	t688	7/62	11
	t008	5/62	8
	t148	4/62	6
	t337	2/62	3
	t189	2/62	3
	t701	2/62	3

t067	2/62	3
t493	2/62	3
t2461	2/62	3
NT	3/62	5
CR Children		
t008	15/59	25
t688	11/59	19
t148	3/59	5
t189	2/59	3
t015	2/59	3
t088	2/59	3
t493	2/59	3
NT	2/59	3

[a]The following spa types occurred only once among: 1) IHO adults: t010, t011, t015, t088, t091, t150, t346, t571, t616, t622, t645, t701, t922, t1077, t1430, t2315, t11124, t14156, t14157; 2) IHO children: t015, t034, t088, t024, t050, t065, t073, t085, t091, t093, t127, t156, t209, t212, t233, t362, t368, t493, t840, t1067, t1937, t3802; 3) CR adults: t002, t012, t015, t034, t065, t088, t089, t091, t164, t185, t216, t223 t330, t338, t346 t645, t1610, t2949, t5859, t7093, t14785; and 4) CR children: t012, t031, t034, t040, t084, t089, t157, t160, t185, t209, t233, t368, t922, t1078, t2461, t2949, t14779, t14781, t14783, t14784.

[b]The total N does not sum to the total number of *S. aureus* nasal carriers because participants who had discordant *spa*-types between their two nasal swab *S. aureus* isolates were not included. This applied to: 1) N=6 adults in IHO group, 2) N=3 children in the IHO group, 3) N=1 adult in CR group, and 4) N=3 children in the CR group. See Table S4 for *spa*-types observed among these participants.

Table S4. *S. aureus spa* types for each participant with discordance in *spa* types in their two nasal swab *S. aureus* isolates[a], North Carolina, 2014.

		S. aureus spa type	
Exposure group	Participant type	Isolate 1	Isolate 2
IHO	Adult	t185	t189
IHO	Adult	t034	t571
IHO	Adult	t085	t1937
IHO	Adult	t156	t233
IHO	Adult	t002	t1937
IHO	Adult	t002	t1937
IHO	Child	t012	t363
IHO	Child	t008	t1885
IHO	Child	t362	t571
CR	Adult	t1911	t338
CR	Child	t034	t089
CR	Child	t017	t622
CR	Child	t644	t14780

[a]We did not observe any households where the adult and child participant both had discordant *S. aureus spa* types.
